# Hybrid Thin-Film Materials Combinations for Complementary Integration Circuit Implementation

**DOI:** 10.3390/membranes11120931

**Published:** 2021-11-26

**Authors:** Gunhoo Woo, Hocheon Yoo, Taesung Kim

**Affiliations:** 1 SKKU Advanced Institute of Nanotechnology, Sungkyunkwan University (SKKU), Suwon 16419, Korea; dnwhddms12@skku.edu; 2 Department of Electronic Engineering, Gachon University, Seongnam 13120, Korea; 3 Department of Mechanical Engineering, Sungkyunkwan University (SKKU), Suwon 16419, Korea

**Keywords:** complementary inverter, thin-film transistors, material integration, organic semiconductors, metal oxides

## Abstract

Beyond conventional silicon, emerging semiconductor materials have been actively investigated for the development of integrated circuits (ICs). Considerable effort has been put into implementing complementary circuits using non-silicon emerging materials, such as organic semiconductors, carbon nanotubes, metal oxides, transition metal dichalcogenides, and perovskites. Whereas shortcomings of each candidate semiconductor limit the development of complementary ICs, an approach of hybrid materials is considered as a new solution to the complementary integration process. This article revisits recent advances in hybrid-material combination-based complementary circuits. This review summarizes the strong and weak points of the respective candidates, focusing on their complementary circuit integrations. We also discuss the opportunities and challenges presented by the prospect of hybrid integration.

## 1. Introduction

Over the past few decades, silicon-based transistor technology has been dominant in the electronics industry because of the excellent electrical characteristics and scaling technology, whereas the limitations of the fabrication process restricted large-scale fabrication and use on flexible substrates [1,2]. Thin-film transistors (TFTs) have been extensively developed with great significance for large-area electronics [3,4,5]. Due to manufacturing advantages, TFTs can be fabricated on a variety of substrates such as flexible plastics [6,7,8], banknotes [9], skin [10,11], and even textiles [12,13,14]. Various TFTs are being explored, targeting flexible (or stretchable) displays [15,16], functional photo- [17,18], gas- [19,20], and bio-sensors [21,22], and healthcare electronics [23,24] as potential applications.

In TFTs, the semiconductor material usually determines the operational type (i.e., *p-*type or *n-*type), where *p-*type TFTs operate via negative gate-source voltage bias when using materials known as *p-*type semiconductors, such as dinaphtho [2,3-b:2′,3′-f]thieno[3,2-b]thiophene (DNTT) [25,26,27], cupric oxide (CuO) [28,29,30], carbon nanotubes [31,32,33,34], and emerging perovskite materials [35,36,37]. In contrast, *n-*type TFTs operate via positive gate-source voltage bias based on materials known as *n-*type semiconductors, such as molybdenum disulfide (MoS_2_) [38,39,40], indium gallium zinc oxide (IGZO) [41,42,43], and N,N`ditridecylperylene-3,4,9,10-tetracarboxylic diimide (PTCDI-C13) [44,45]. As noted and listed above, fabrication process combinations of different materials are required to build complementary circuitry, composed of the respective *p-*type and *n-*type TFTs.

A complementary metal oxide semiconductor (CMOS) is based on a field-effect transistor (FET) manufacturing process that uses complementary and symmetric pairs of *p*-type and *n*-type FETs. CMOS is built with a combination of a *p-*type transistor and an n-type transistor, and switching the on/off state of the *p*-/*n*-transistor along with sweeping the input voltage produces a change in the signal corresponding to “0” and “1” states. A CMOS inverter circuit has the *n*-/*p*-transistors connected to drain and gate electrodes, a supply voltage (V_DD_) to the pull-up *p*-transistor, and ground (0 V) connected to the pull-down *p*-transistor. The performance of a CMOS inverter is determined by the following parameters: (1) voltage gain, (2) noise margin (the amount of the noise of the output voltage that is affordable to withstand the operation failure), and (3) power consumption. Since the symmetrically matched *I_D_-V_D_* curve of an *n-*/*p-* transistor can accompany the excellent inverter behavior, modulating the shape of the *I_D_-V_D_* curve of each transistor is the crucial factor. Furthermore, introduction of a high-k dielectric layer is useful to reduce the power consumption by reducing the operation input voltage range [46].

For complementary technology, as a general approach, transistors are made using the same material family, i.e., small molecules, polymers, oxides, and transition metal dichalcogenides (TMDs). Another approach was attempted by implementing complementary transistors using more than one different material family. Implementing complementary behavior using these heterogeneous materials makes a hybrid complementary TFT. This hybrid complementary approach offers advantages that compensate for the disadvantages of each family of materials. Low temperature polysilicon oxide (LTPO) CMOS is proper example to explain the possibility of a hybrid CMOS inverter. The combination of the high mobility property from a *p*-channel low temperature polysilicon (LTPS) TFT and the low off-current property from an *n*-channel *a*-IGZO TFT led to enhanced device performance [47]. Furthermore, the hybrid materials combination approach can accompany the simplification of the fabrication process. For example, polymer-based patterning processes are mainly conducted to modify the dimension of TMD materials, causing defects during the etching process or material contamination. In contrast, metal oxide or organic materials are simply patterned with a shadow mask or inkjet printing method, which reduces the complex patterning process [48].

In this context, we summarize here the recent progress in the development of hybrid complementary TFTs. This review is motivated by the emergence of various thin-film semiconducting materials enabling next-generation functionality in TFTs. This review provides an overview of the recent contributions to hybrid complementary integrations using emerging TFT materials: (1) 2D TMDs, (2) metal oxides, (3) organic semiconductors, (4) perovskite materials, and (5) carbon nanotubes. Furthermore, we introduce a newly investigated approach to heterogenous hybrid TFTs, presenting novel applications to multi-valued logics and vertically stacked inverters.

## 2. Materials for Hybrid Inverters

### 2.1. Two-Dimensional Transition Metal Dichalcogenides Materials

**Figure 1:**
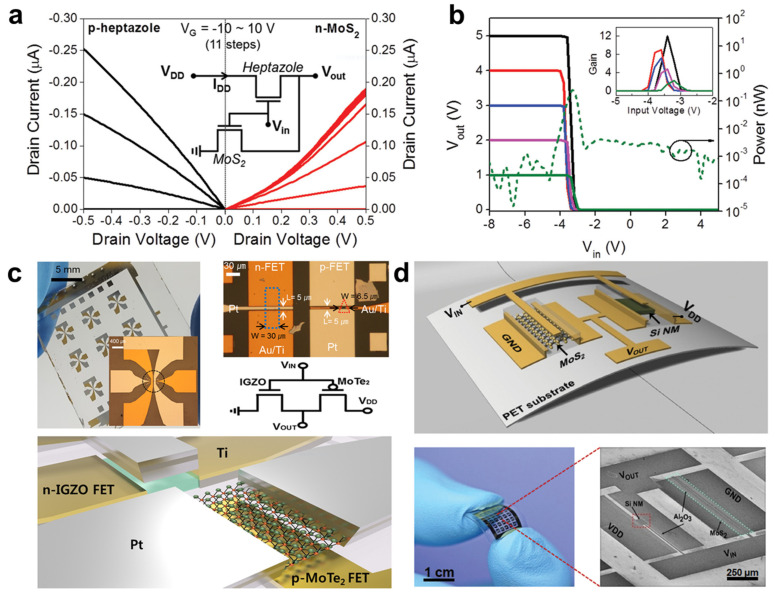
(**a**) *I*_D_–*V*_D_ output curve of *n-*MoS_2_ and *p-*heptazole channel FETs. (**b**) The voltage transfer characteristics of a hybrid CMOS inverter under supply voltages of 1 V to 5 V. The dashed curve shows power consumption at 1 V (adapted from [63] with permission from John Wiley and Sons). (**c**) Patterned CMOS inverter arrays, an OM image of the hybrid inverter fabricated on glass, and a 3D schematic of the hybrid CMOS inverter (adapted from [64] with permission from the American Chemical Society). (**d**) A 3D illustration of an Si NM-MoS_2_-based complementary inverter built on a plastic substrate, and a photographic image of a large-area 5 × 5 array of a CMOS inverter patterned on PET, where the magnified panel shows the scanning electron microscope (SEM) image (adapted from [65] with permission from John Wiley and Sons).

Two-dimensional (2D) TMD material comprises multiple stacked layers with thicknesses in sub-nanometers [49,50,51]. The 2D structure aligns the electron transportation to the plane while constraining the Z-axial direction, and each layer is coupled by a weak van der Waals (vdWs) force. The structural specificity produces superior electrical and optical properties, and induces a sharp contact condition with other materials [52,53]. Moreover, because of their excellent mechanical properties such as flexibility and rigidity, 2D materials have attracted a lot of attention as potential candidates for various applications such as field-effect transistors (FET) [54,55], gas sensors [56], photo sensors [57,58] and flexible or ubiquitous substrates [59,60]. The information of electrical properties and structure dimension of 2D TMD materials was tabulated in Table 1. However, preparation of the high-quality TMD materials for large-scale applications is still limited because of obvious pros and cons for each method: (1) Exfoliation (pros—easy-to-fabricate process, cons—only for flake-scale device), (2) hydrothermal synthesis (pros—mild synthesis condition and layer-scale film, cons—low uniformity), and CVD growth (pros—good quality material with large-scale, cons—limitations in usage flexible substrate) [61,62]. In 2015, Lee et al. reported a hybrid CMOS logic inverter that is built with a top gate *n-*MoS_2_ nanosheet and a bottom gate *p-*heptazole FET [63]. To match the *I_D_-V_D_* curve of the *n-*channel MoS_2_ FET symmetrically to that of the *p-*channel heptazole FET, the threshold voltage, mobility, and drain current level were modified by controlling the thickness of the MoS_2_ layer and inserting a CYTOP buffer layer between the MoS_2_ and insulate layers (Figure 1a). The fabricated organic–inorganic hybrid inverter exhibited a significant voltage gain of 12 *v*/*v* at a supplied voltage of 5 V with only a few hundred picowatts (pW) in power consumption (Figure 1b). In 2017, Lee et al. fabricated a 2D nanosheet-oxide film hybrid inverter comprising a *p-*channel MoTe_2_ FET and an *n-*channel IGZO FET, as shown in Figure 1c [64]. By using a 2D TMD semiconductor that possesses favorable optical and electrical properties for inverter applications, the author achieved significant voltage gain as high as 40 *v*/*v* at a drain voltage of 5 V with only a few nanowatts (nW) in scale power consumption. In 2016, Das et al. reported a highly flexible and large-scale Si-MoS_2_ hybridcomplementary inverter combining an *n-*channel MoS_2_ FET and a *p-*channel Si nanomembrane (NM) FET [65]. By transferring the CVD-grown MoS_2_ and the Si NM, which showed proper flexibility on a flexible polyimide substrate, a bendable Si-MoS_2_ hybrid complementary inverter was implemented (Figure 1d). The voltage transfer curve of the *n-*MoS_2_ and *p-*Si NM hybrid inverter showed a voltage gain of 12 *v*/*v* at a 5 V supply voltage with low power consumption of 100 nW. In addition, the performance of the flexible hybrid inverter was maintained under bending situations. During the variation of the bending radius (3.2 nm, 4.0 nm, 5.4 nm, 6.2 nm, flat), the maximum voltage gain and threshold voltage were 10.75 ± 2 *v*/*v* and 2.34 ± 0.30 V, respectively. The normalized voltage gain within 5% and 20% and high consistency of the threshold voltage during 100 bending cycles showed the performance reliability of the proposed hybrid inverter.

### 2.2. Metal Oxide Semiconductors

**Figure 2:**
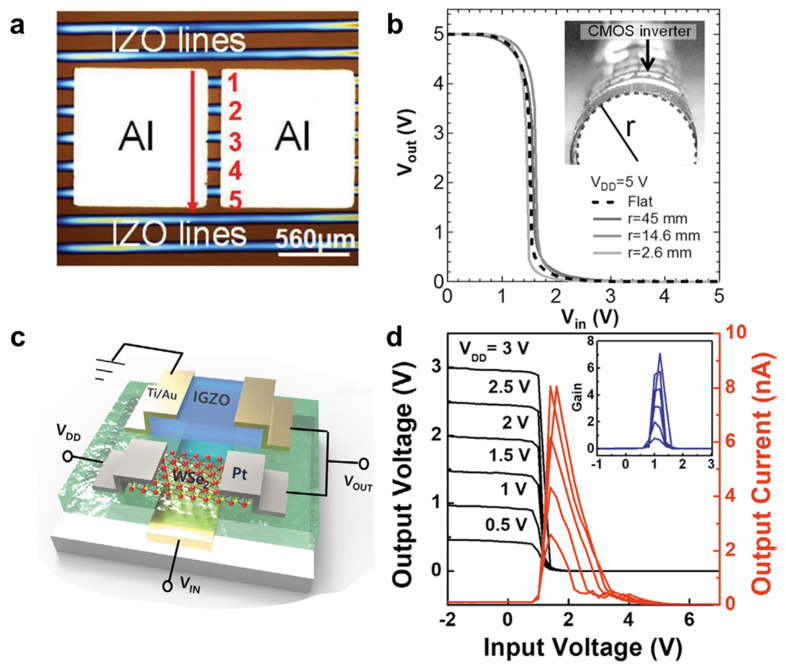
(**a**) Top view OM image of an IGZO TFT fabricated with an inkjet printing method (adapted from [80] with permission from John Wiley and Sons). (**b**) Output voltage characteristics of a 3D CMOS inverter allowing a bending radius up to 2.6 mm (adapted from [83] with permission from John Wiley and Sons). (**c**) A 3D illustration of a CMOS inverter consisting of *p-*WSe_2_ and *n-*IGZO FETs fabricated on a glass substrate. (**d**) Voltage transfer characteristic curves (black) with voltage gain (inset) and output current (red) of proposed CMOS inverter as obtained from 0.5 to 3 V of *V_DD_* (adapted from [84] with permission from John Wiley and Sons).

Metal oxide semiconductors provide many opportunities in various application areas based on their great electrical properties and simple synthesis methods [72,73]. Because the large metal ns orbital of metal oxide sufficiently overlaps an adjacent metal s orbital without significant influence on the existing oxide [74], the metal oxide can maintain high electrical performance regardless of its shape and the bending of the material [75]. Additionally, metal oxide has a very mild synthesis condition, which is highly compatible with applications on a flexible substrate such as polyethylene terephthalate (PET) and polyimide (PI) [75,76]. However, the formation energy of the native acceptors is higher than that of the native donors such as oxygen vacancy, resulting in constraint on the hole generation. Further, the strong localization of the valence band maximum (VBM) to oxygen ions leads to a large hole effective mass and low mobility [77,78,79]. For this reason, most metal oxide semiconductors are an *n-*type material, and even *p-*type metal oxide semiconductors such as CuO, SnO, and NiO show poor charge mobility [80]. Moreover, because most of the low-temperature deposition techniques of metal oxide semiconductors have mainly considered *n*-type materials (for example, indium oxide (In_2_O_3_), indium zinc oxide (IZO), and IGZO), efforts to implement *p*-type metal oxide devices on flexible substrates are still lacking [81,82]. In 2019, Luo et al. implemented a low-voltage, high-performance complementary inverter with an *n-*channel IZO TFT and a *p-*channel chirality-enriched (9,8) semiconducting single-walled carbon nanotube (SWCNT) FET using a partial printing method [80]. Inkjet printing is a highly fascinating process for fabricating TFTs or integrating CMOS circuits because of customizability based on patterned deposition and a simple patterning process for a large-scale array. The author introduced IZO, a type of metal oxide, as a candidate for ink material because of its excellent electrical characteristics and environmental stability (Figure 2a). The hybrid CMOS inverter presented a high voltage gain of 45 *v*/*v* with low-power consumption of 400 nW. Moreover, based on a well-matched *p-*TFT and *n-*TFT, all noise margins showed more than 0.73 V (~73% of the 1/2 *V_DD_*). In 2015, Honda et al. demonstrated a temperature-response, vertically integrated CNT and IGZO hybrid inverter, and evaluated it by integrating the temperature sensor on a third layer [83]. The IGZO and CNT TFT were deposited on the first and second polyimide layers, and each layer was stacked vertically. The proposed vertical hybrid inverter showed a good voltage gain of 45 *v*/*v* with 6.9 nW mm^−1^ low-power consumption. Mechanical flexibility in the vertical hybrid inverter was also evaluated by bending it, depending on the curvature radius, from flat to 2.6 mm. The peak gain and the threshold voltage of the hybrid inverter exhibited a uniform level of value, regardless of the curvature radius, and showed stable performance and durability even in the 1000-cycle bending test, as shown in Figure 2b. The *I_DS_-V_GS_* curves of the CNT and IGZO TFT changed in proportion to the temperature variation, implementing a temperature-response hybrid inverter. As temperature increased, the threshold voltage of the hybrid inverter decreased linearly. In 2020, Lee et al. integrated a hybrid inverter using *n-*IGZO and *p-*WSe_2_ applied to two circuit models [84]: (1) a complementary metal oxide semiconductor with an *n-*channel IGZO and a *p-*channel WSe_2_ (Figure 2c,d), and (2) a heterojunction *p-*WSe_2_/*n-*IGZO diode-load inverter. Introducing a WSe_2_/IGZO diode instead of a WSe_2_ TFT increased the voltage gain from 6.5 *v*/*v* to 14 V/V, and low power consumption of just a few nanowatts was achieved. Moreover, the photo-response characteristic of the WSe_2_/IGZO diode allowed variations in output voltage under illumination.

### 2.3. Organic Semiconductors

**Figure 3:**
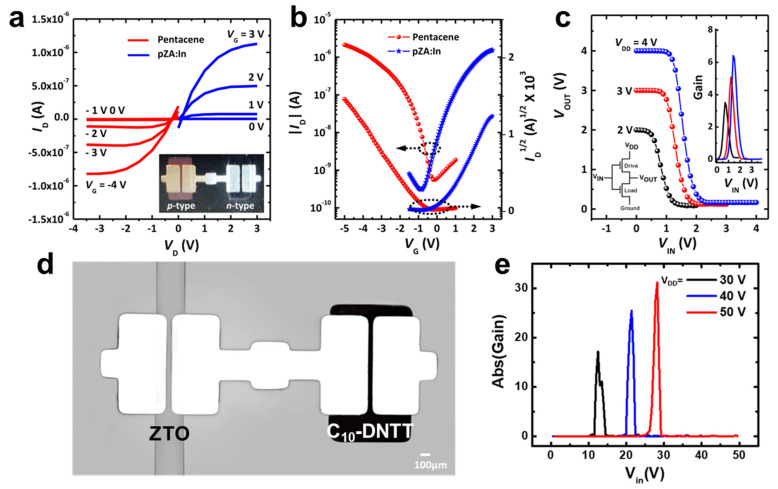
(**a**) Transfer characteristics of ZnO TFTs depending on indium doping. (**b**) Transfer characteristics and (**c**) output voltage characteristics of the pentacene and pZA:In-ZnO TFTs (adapted from [46] with permission from the American Chemical Society). (**d**) Top view OM image of a hybrid inverter comprising a printed ZTO transistor and a C_10_-DNTT transistor. (**e**) The voltage gains in the CMOS hybrid inverter under three *V_DD_* conditions (*V_DD_* = 30, 40, and 50 V) (adapted from [92] with permission from MDPI).

Organic semiconductors have many advantages such as flexibility, lightness, and elaborate control of material properties through molecular structure control. For this reason, organic semiconductors are being actively utilized in applications such as flexible/wearable displays and bio or chemical sensors. [85,86,87,88]. Moreover, because organic semiconductors (i.e., pentacene, fused aromatic compound, and rubrene) are representative *p-*type material, combination with the 2D material or oxide semiconductor can produce significant synergistic effects [89,90]. However, many studies are still in progress to improve their limited electrical properties, vulnerability to temperature, and instability in the surrounding environment [91]. In 2016, Jeong et al. introduced a photocurable polymer precursor, zinc diacrylate (ZDA), to fabricate a patternable organic/inorganic hybrid inverter [46]. Organic- and metal oxide-based semiconductors are highly promising candidates because of their low fabrication cost and simple patterning process. In a commercial application of a solution processed ZnO, the author noticed two requirements for integrating a circuit: (1) a simple patterning process without photolithography, and (2) low operating voltage for device operational stability. This report presented zinc diacrylate-enabled synthesis of a patterned ZnO with a simple UV polymerization and annealing process. Pentacene was utilized as a *p-*type organic semiconductor in the organic/inorganic hybrid inverter. By using high dielectric film, Al_2_O_3_/TiO_2_, to reduce the operation voltage, both organic and inorganic TFTs exhibited great output (Figure 3a) and a transfer characteristic (Figure 3b) under low operation voltages from −5 to 3 V. Moreover, the hybrid inverter showed a voltage gain as high as 6.5 *v*/*v* (Figure 3c). In 2020, Ye et al. introduced a highly functional inkjet printing method that can print zinc-tin oxide (ZTO) at a high-resolution nanoscale assisted by an applied electrostatic field [92]. By using electrohydrodynamic (EHD) inkjet printing, a ZTO TFT was fabricated as an *n-*channel semiconductor after a simple annealing process, whereas C_18_-DNTT was utilized as a *p-*channel semiconductor for the organic/inorganic hybrid inverter (Figure 3d). The resulting inverter demonstrated a high voltage gain of over 30 *v*/*v* at a drain voltage of 50 V (Figure 3e).

### 2.4. Metal-Halide Perovskite

**Figure 4:**
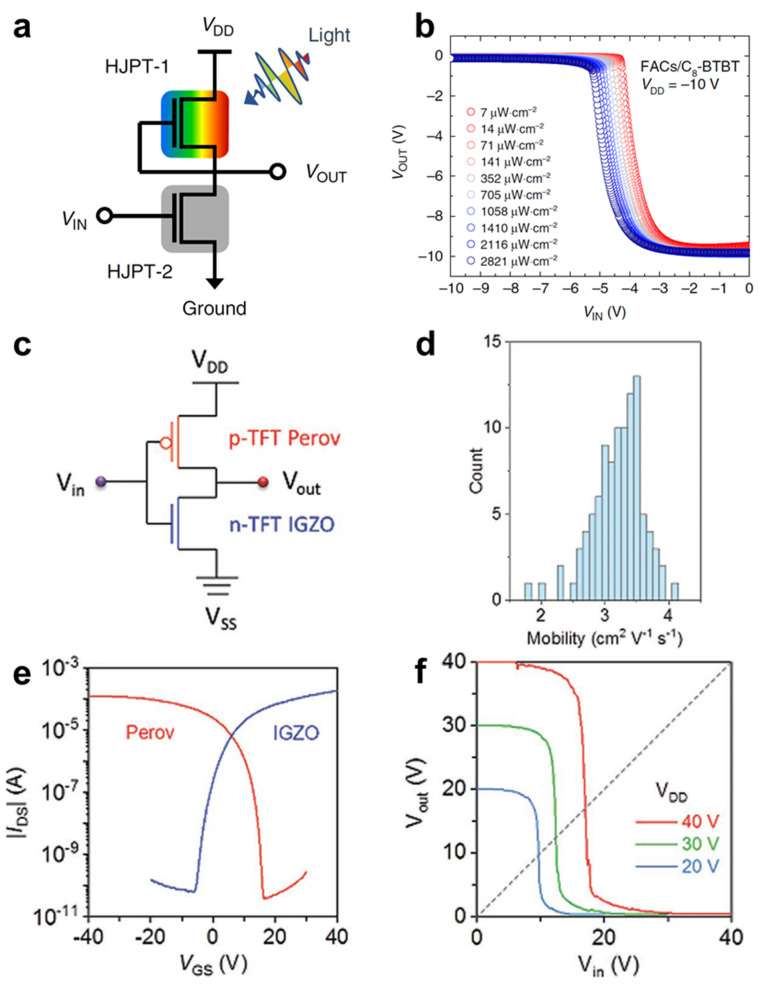
(**a**) Transfer characteristics for heterojunction phototransistor (HJPT) photo-inverters. (**b**) Transfer characteristics measured using a 475 nm LED light source under different incident light intensities for photo-inverters composed of HJPTs using FACs/C8-BTBT (adapted from [98] with permission from Springer Nature). (**c**) Circuit diagram of a (PEA)_2_SnI_4_ TFT array on a four-inch Si/SiO_2_ (100 nm) wafer substrate. (**d**) Statistical distribution of mobility obtained from 100 TFTs across the array. (**e**) Transfer characteristics of perovskite and IGZO TFTs. (**f**) VTC of a complementary inverter at different *V_DD_* values (adapted from [99] with permission from John Wiley and Sons).

Metal-halide perovskites (MHPs) have been mostly utilized in photoelectric applications like solar cells and light-emitting diodes because of their strong absorption coefficient and tunable optical bandgap [93,94,95]. MHP is an especially good *p-*type semiconductor, and an excellent counterpart to an *n-*type inorganic semiconductor [96,97]. We additionally provided electrical characteristics of representative *p-*type organic semiconductors and perovskites in Table 2. However, satisfying good quality in the semiconductor material and for mass production is still difficult, and ion migration makes MHP insensitive to gating [98]. For this reason, many attempts are still being made to fabricate MHP-based electronics. In 2019, Len et al. introduced a straddling-gap (type-I) organic semiconductor/metal-halide perovskite heterojunction to obtain state-of-the-art photogain of 15 *v*/*v* and tunable photoresponsivity [98]. Straddling-gap (type-I) indicates that the bandgap of one semiconductor (in this case MHP) is completely included in that of another semiconductor (in this case organic semiconductor). Type-I FA_0.83_Cs_0.17_PbI_2.7_Br_0.3_ (FACs)/C_8_-BTBT heterostructure makes it easy to preserve the hole majority of the photocarriers at the valence band of the MHP. The preserved photocarriers were transported under the regime of off-state of the FAC/C_8_-BTBT heterojunction phototransistors (HJPT) and changed the on/off current ratio. To compare the photoresponsivity along with band structure, the author investigated type-II FAC/C_16_-BTBT HJPT, and Type-I FAC/C_8_-BTBT HJPT exhibited photoresponsivity at off-state. To access performance of the proposed phototransistor, a PMOS-like photo-inverter was fabricated using two of the same FAC/C_8_-BTBT HJPTs. When illuminated on a HJPT 1, as shown in Figure 4a, the voltage transfer curve showed a large output voltage of −10 V at the lowest intensity of light. The maximum voltage gain remained at a relatively unchanged value of 15 *v*/*v* regardless of light intensity, but the input voltage at maximum amplification (A_i,max_) was shifted from −5 to −4 V as the light intensity increase from 7 to 2821 μW·cm^−2^ (Figure 4b). In 2020, Zhu et al. synthesized a highly reliable, lead-free perovskite-based TFT and investigated the feasibility of *p-*type transistor as an inverter application (Figure 4c) [99]. The stubborn ion migration, making it insensitive to the applied bias, inhibited the use of perovskite as the active material for transistors. The author overcame poor synthesis quality with the following solutions: (1) grain boundary passivation using extra PEAI, (2) introduction of Sn powder to reduce oxidation of the Sn precursor, and (3) grain crystallization engineering through the addition of Lewis bases. As a result, the proposed (PEA)_2_SnI_4_ TFT achieved good electrical performance (mobility = 3.5 cm^2^/V· s, on/off ratio = 3.4 × 10^6^) in *p-*type current behavior. The perovskite-based complementary inverter was fabricated using an *n-*type IGZO TFT and *p-*type (PEA)_2_SnI_4_ TFT, which showed a large gain of 30 *v*/*v* with a high noise margin of 70% at V_DD_ of 14 V, and the performance reliability was confirmed through 100 devices evaluation (Figure 4d–f).

### 2.5. Carbon Nanotubes

**Figure 5:**
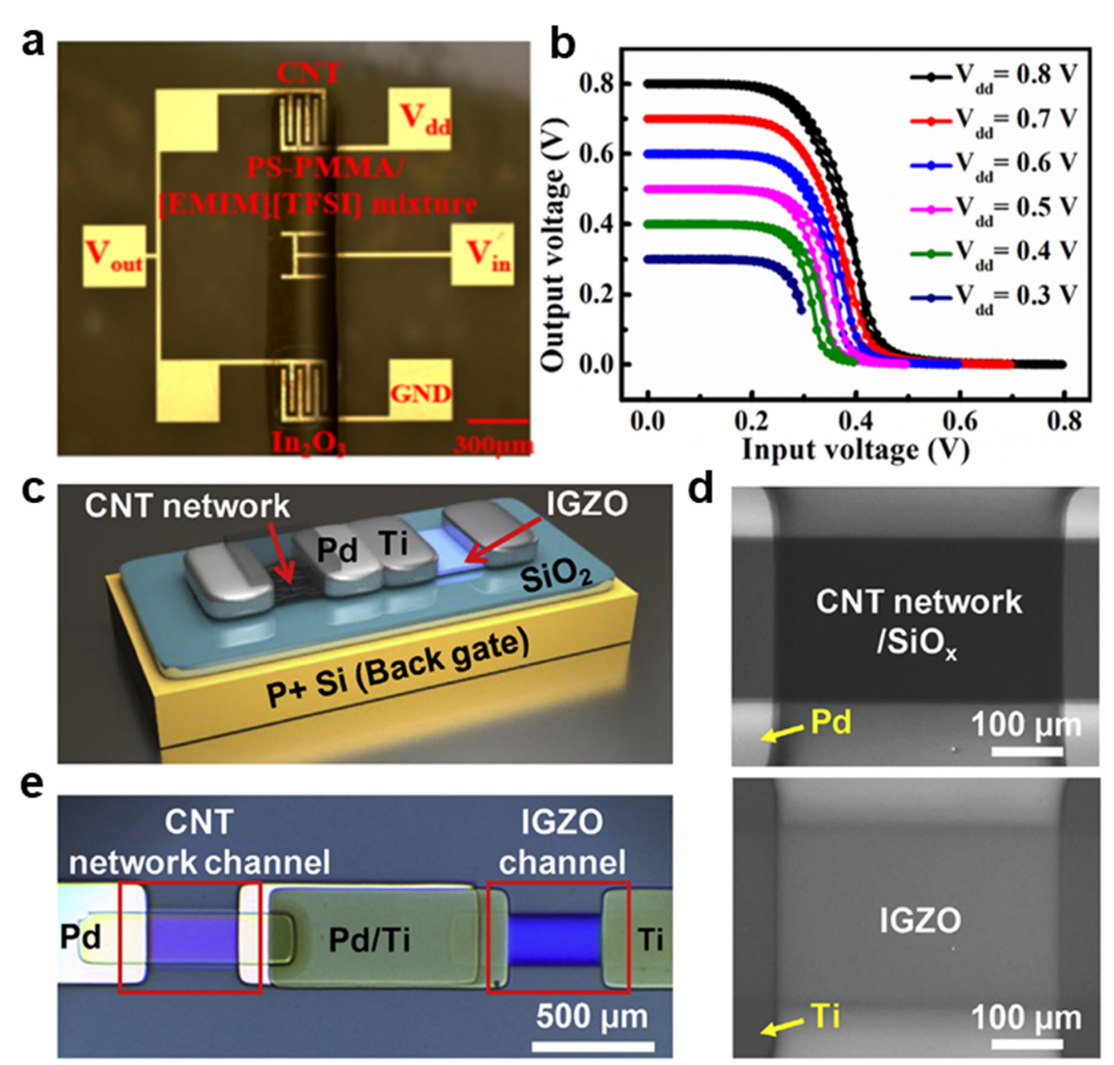
(**a**) Structure of the printed *p-*CNT and *n-*In_2_O_3_ channel hybrid CMOS inverters. (**b**) Voltage transfer characteristics of the proposed printed hybrid CMOS inverter (adapted from [109] with permission from the American Chemical Society). (**c**) Structure schematic of the hybrid complementary inverter composed with *p*-type CNT and *n*-type IGZO TFTs. (**d**) SEM images of the CNT network channel and IGZO channel. (**e**) Optical microscope (OM) image of the hybrid complementary inverter (adapted from [110] with permission from Elsevier B.V.).

The carbon nanotube (CNT) is a highly promising *p-*type semiconductor material because of its outstanding electrical and optical properties, which originate from a unique one-dimensional structure [104]. Moreover, the inkjet printing method can produce a patterned CNT on any kind of substrate, and much research has achieved remarkable accomplishments in fabricating CNT FETs [105,106]. Combination with an *n-*type material can extend the opportunity for applying the CNT *p-*type transistor to a CMOS inverter. Nevertheless, the practicality of CNTs for CMOS inverters still needs further improvement to resolve the inadequate air stability and limited tunability [107,108]. In 2020, Luo et al. manufactured a radiation-hard and repairable complementary hybrid inverter using a simple inkjet printing method [109]. The CNT and In_2_O_3_, which were regarded as favorable candidates for printable conducting materials, were used as *p-*type and *n-*type transistors for the proposed hybrid inverter (Figure 5a). Additionally, the PS-PMMA/[EMIM][TFSI] covered two transistors as the gate dielectric layer and served to passivate from the strong radiation. The hybrid inverter showed ultralow power consumption of 9.7 μW at a drain voltage of 0.8 V. Moreover, the voltage gain increased to 11.5 *v*/*v* and a large noise margin of 75% was exhibited (Figure 5b). To confirm stability under radiation, probing of the voltage transport curve was conducted, depending on an intensity of the Co-60 γ irradiation. In 2018, Yoon et al. demonstrated an optimization process of hybrid integration of a *p-*type carbon nanotube TFT and an *n-*type IGZO TFT (Figure 5c–e) [110]. Because the CNT and IGZO are representative *p-*type and *n-*type materials, respectively, the difficulty in manufacturing a homogenous complementary inverter using only the CNT or IGZO was resolved by integrating a hybrid inverter. In this regard, the author optimized the fabrication condition of the complementary microelectronic circuits by adjusting the CNT deposition time and the oxygen flow rate during IGZO sputtering. The proposed hybrid inverter exhibited an optimized voltage gain of 108 *v*/*v* at the oxygen flow rate of 0.1 sccm and CNT deposition time of 5 min.

## 3. Applications

### 3.1. Multivalued Logics

**Figure 6:**
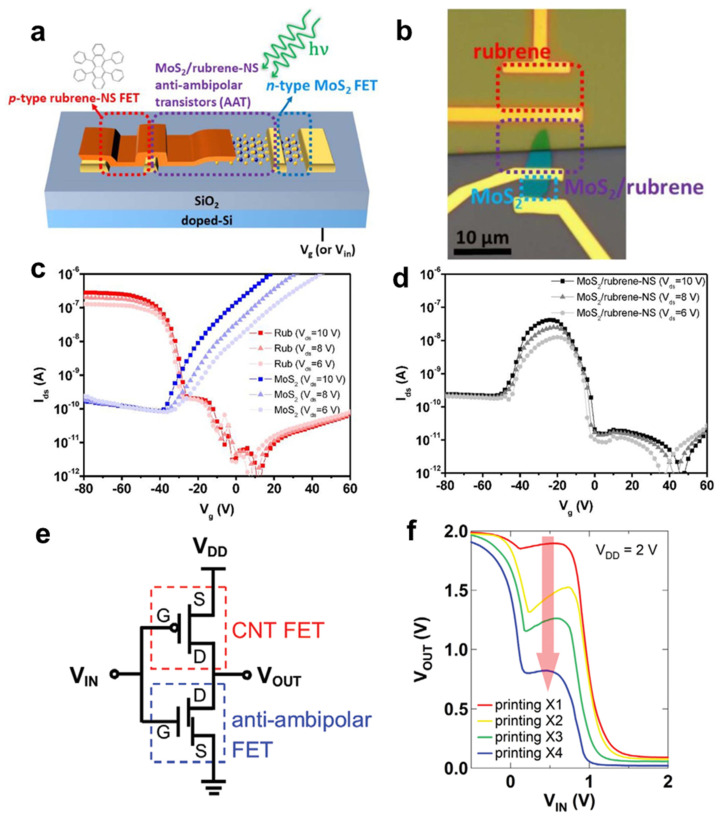
(**a**) Illustration of a ternary inverter using a lateral-type MoS_2_/rubrene-NS *n-p* heterojunction FET. (**b**) Optical microscope image of the ternary inverter using the lateral-type MoS_2_/rubrene-NS heterojunction FET. (**c**) Transfer characteristic curves of the rubrene-NS–based FET and the MoS_2_-based FET. (**d**) Transfer characteristic curves (*I_DS_–V_GS_*) of the MoS_2_/rubrene-NS *n-p* heterojunction FET (AAT) (adapted from [113] with permission from IOP Publishing). (**e**) Circuit diagram of the ternary inverter composed of a CNT FET and an anti-ambipolar FET. (**f**) Voltage transfer characteristics of the ternary inverter (adapted from [114] with permission from John Wiley and Sons).

By employing various semiconductors that possess different types of major carriers with each other, the CMOS has overcome many limitations in terms of fabrication processes and device performance enhancement. Furthermore, in some trials, an *n-p* heterojunction transistor was utilized instead of one of the transistors in the CMOS, which showed a reversed ambipolar *I-V* curve [26,111]. The integrated inverter produces the third state of “1/2” excluding “1” and “0” at the voltage transport curve, which is called a ternary inverter. Study of the ternary inverter is highly meaningful because of the high density of the data and simplification of the circuit system [112], and there were attempts to implement a hybrid ternary inverter. In 2020, Park et al. reported a photo-triggered ternary inverter using a rubrene nanosheet (NS) TFT and a rubrene/MoS_2_ *n-p* heterojunction anti-ambipolar transistor (AAT) (Figure 6a,b) [113]. A largely unmatched threshold voltage of the MoS_2_ and rubrene NS created a wide on-state voltage range in the middle of the voltage sweeping range, which formed an anti-ambipolar shape in the *I-V* curve (Figure 6c,d). Interestingly, the proposed AAT/rubrene NS hybrid inverter selectively showed ternary inverter behavior under a specific wavelength of light. Under illumination at 455 nm and 530 nm wavelengths, the threshold voltages of the MoS_2_ (and especially the rubrene NS) shifted, which extended the voltage range of the on-state in the AAT. The voltage range variation in the AAT induced the third state, “1/2”, in the photo-responsive ternary inverter. In 2020, Kim et al. fabricated a fully printable ternary inverter by employing a *p-*type CNT TFT and an indium oxide/CNT *n-p* heterojunction–based AAT (Figure 6e) [114]. Since the dimensions of the semiconductor significantly influence performance of the transistor, the number of printings in the inkjet printing method is a crucial parameter for deciding device performance. Therefore, the author modified the on-state range of the AAT by controlling the number of printings, and clearly optimized the ternary inverter behavior at four printings. As shown in the voltage transfer curve (Figure 6f), the output voltage value at the “1/2” state nearby V_in_ = 0.5 V was gradually changed through an increase in the number of printings. Further, by applying a three-valued input signals at V_in_ = 0, 1, and 2 V, the dynamic operation of the proposed ternary inverter was investigated, and the result demonstrated clear output signals at 0.21, 0.05, and 0 V, respectively. It is noted that the further advanced ternary circuit such as two-stage cascaded circuit was implemented [115].

### 3.2. Vertically Stacked Complementary Inverter

**Figure 7:**
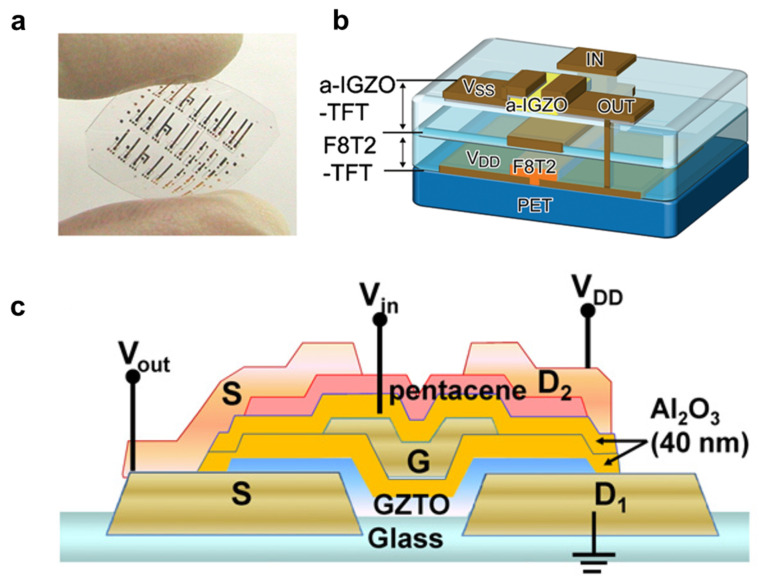
(**a**) Image of the flexible hybrid inverter comprising *n*-type IGZO TFT and *p*-type F8T2 TFT and that of (**b**) the device structure (adapted from [122] with permission from American Institute of Physics). (**c**) Illustration of a cross-section view of the Vs-CTFT inverter with *n*-type GZTO TFT and *p*-type pentacene TFT (adapted from [118] with permission from Elsevier B.V.).

To enhance the density of data in a single pixel, not only to increase number of logic values, many studies also have tried to improve by structural modulation by stacking the complementary inverter vertically [116,117]. Their three-dimensional (3D) stacked structure can minimize the physical distance and increase the drivability of the electronic circuits by resolving the interconnection lengths and parasitic resistances [118,119]. However, the fabrication process of the vertically stacked inverter is highly complicated and difficult [120,121]. In 2010, Nomura et al. fabricated vertically stacked *n*-type IGZO transistors and *p*-type poly-(9,9-dioctylfluorene-co-bithiophene) (F8T2) thin film transistors on a flexible PET substrate as shown in Figure 7a,b [122]. Both transistors demonstrated low off-current and huge on/off current ratio, and the positions of the threshold voltage of each transistor certified the well-matched *I_DS_-V_GS_* characteristic of the *p*-F8T2 TFT and *n*-IGZO TFT. The voltage transfer characteristic of the proposed vertical hybrid inverter showed gain as high as 67 V/V. The high noise margin and low noise margin were 2.1 and 6.3 V at *V_DD_* = 10 V and 10.4 and 18.3 V at *V_DD_* = 30 V. In 2011, Park et al. implemented vertically stacked organic/oxide hybrid inverter by using *p*-channel pentacene TFT and *n*-channel GaZnSn oxide (GZTO) TFT, as shown in Figure 7c [118]. The author investigated not only performance as a function of inverter but also operation in photogating and ferroelectric memory. The proposed hybrid vertical inverter exhibited clear inverter operation with high voltage gains of 20, 25, and 52 *v*/*v* at supply voltage of 3, 5, and 8 V, respectively. Moreover, the hybrid inverter operated with a response time of 5–40 ms under 5 V input pulse. The response time of the proposed hybrid complementary inverter was yet not comparable with the commercial product, so further efforts to improve device performance are still necessary [123].

## 4. Conclusions and Outlook

In this review, we present an overview of recent advances in hybrid-material combination-based complementary circuits. Non-silicon materials are considered highly promising material because their unique material properties help to implement CMOS inverters, which obtain various functionality such as flexibility, transparency, and so on. However, their intrinsic characteristics compromise the CMOS inverter by using similar types of materials. Hybrid inverters provide solutions to many problems and allow implementation of functional inverter devices. We listed five non-silicon semiconductors and summarized the pros and cons of using them in hybrid inverters.

(1) Two-dimensional materials possess a layered structure based on van der Waals force, which assigns excellent electrical performance, high material stability in the surrounding environment, and great mechanical properties. However, their unique structure is mainly implemented with specific synthesis conditions, confining the compatibility with certain kinds of substrate.
(2) Metal oxide semiconductors are highly promising because of their mild synthesis condition, ease of fabrication for large-scale applications, and great electrical performance. However, most metal oxide semiconductors are an *n-*type material, and even *p-*type metal oxide semiconductors show poor charge mobility and a high annealing temperature.
(3) Most organic semiconductors exhibit *p*-type characteristics, unlike 2D materials and oxide semiconductors, which are very important to fabricate CMOS inverters. Moreover, their simple synthesis process allows them to be fabricated on flexible devices. However, vulnerability to temperature and instability in the surrounding environment still remain a challenge to overcome.
(4) Strong absorption coefficients and tunable optical bandgaps of MHPs contribute to optoelectrical applications. Specifically, MHP is a good *p-*type semiconductor, which is highly compatible to fabricate hybrid inverter with an *n-*type inorganic semiconductor. Nevertheless, the practicality of perovskites for CMOS inverters still needs further improvement for good electrical quality and mass production.
(5) CNT have attracted attention for their printable synthesis method and *p*-type semiconductor characteristics. However, using CNT is still limited because of their inadequate air stability and limited tunability.

Material characteristics and preparation process of each type of semiconductor materials and their inverter performances were listed up in Table 3 and Table 4.

Complementary inverters possess significant potential for application to not only logic components but also various sensors (such as chemical sensors [124,125], optical sensors [126], gas sensors [127], and temperature sensors [83]) and biomedical applications (such as bioelectronics [128,129] and bio-signal amplifiers [119,130]). However, challenges for integrating hybrid materials still exist; the fabrication process of *p*-type and *n*-type materials and their devices requires the separate deposition, patterning, and optimization of two heterogenous materials, increasing the complexity of the fabrication process with the ad hoc process conditions. Therefore, it is important to deeply understand the material intrinsic properties and discover the desirable integration process of the hybrid materials combination, and this paper is expected to provide useful guidelines for dealing with hybrid complementary integrations.

## Figures and Tables

**Table 1:** Electrical characteristics and structure dimensions of 2D TMD material.

**Materials**	**Conduction Type**	**Mobility (cm^2^/V·s)**		**Band Gap (eV)**	
		**Multilayers (>10 Layers)**	**Monolayer**	**Multilayers (>10 Layers)**	**Monolayer**
2H-MoS_2_	*n*-type	60–200	>200	1.23	1.89
2H-MoSe_2_	*n*-type	160–260	50	1.09	1.57
2H-MoTe_2_	*p*-type	40	N/A	0.93	1.08
2H-WS_2_	*n*-type	20–100	0.2	1.35	1.98
2H-WSe_2_	*p*-type	120–150	30–180	1.20	1.66
1T’-WTe_2_	N/A	6000–44,000	20–21,000	Semimetal/metal	
**Materials**	***^α^*** **Interlayer Distance (** **Å** **)**	***^β^*** **vdW Gap (** **Å** **)**	**MX_2_ Sandwich Thickness (** **Å** **)**	**M-X Bond Length (** **Å** **)**	***^γ^*** **M|M Distance (** **Å** **)**
2H-MoS_2_	6.15	2.98	3.17	2.42	3.16
2H-MoSe_2_	6.47	3.24	3.23	2.49	3.29
2H-MoTe_2_	7.28	3.68	3.60	2.72	3.52
2H-WS_2_	6.16	3.02	3.14	2.40	3.15
2H-WSe_2_	7.00	3.76	3.24	2.49	3.29
1T’-WTe_2_	7.02	3.80–3.90	3.50–4.00	2.71–2.82	2.86

*^α^* Distance the M atomic planes in two neighboring layers. *^β^* Closest distance between the X atomic planes in two neighboring layers. *^γ^* Closest distance between two M atoms (also between two X atoms). Data collected from the following references: [66,67,68,69,70,71].

**Table 2:** The electrical properties of perovskite and organic semiconductors.

Materials	Conduction Type	On/off Current Ratio	Mobility (cm^2^/V·s)	Subthreshold Swing (V·dec^−1^)	Threshold Voltage (V)	Ref.
(PEA)_2_SnI_4_	*p*-type	3.4 × 10^6^	3.51	0.8	7.3	[99]
MAPbI_3_	*p*-type	2.5 × 10^4^	23.2	0.14	−0.57	[100]
C_10_-DNTT	*p*-type	10^8^	4.3	68	−0.4	[101]
DNTT	*p*-type	10^8^	2.1	100	−1.4	[102]
Pentacene	*p*-type	10^7^	0.8	75	−0.6	[103]

**Table 3:** Performance comparison of the hybrid complementary inverter.

*n-*Type Material	*p-*Type Material	NMOS Mobility (cm^2^/V·s)	PMOS Mobility (cm^2^/V·s)	Voltage Gain (*V*/*V*)	Noise Margin	Operation Voltage (V)	Power Consumption (nW)	Ref.
MoS_2_	Heptazole	6	0.14	12	N/A	5	1	[69]
IGZO	MoTe_2_	4.2	22.4	40	N/A	5	300	[70]
MoS_2_	Si NM	N/A	N/A	16	^α^ NM_T_ 80%	5	300	[71]
IZO	SWCNT	3.01	3–5	45	^β^ NM_H_ 77% ^γ^ NM_L_ 83%	2	400	[80]
IGZO	CNT	4.93	2.19	45	N/A	5	0.69	[83]
IGZO	WSe_2_	N/A	N/A	6.5	N/A	3	N/A	[84]
pWA:In-ZnO	Pentacene	0.853	0.718	6.5	N/A	4	N/A	[46]
ZTO	C_10-_DNTT	1.35	N/A	31.2	N/A	50	N/A	[92]
FACs/C_8_-BTBT	FACs/C_8_-BTBT	0.52	0.52	15	N/A	−10	N/A	[98]
IGZO	(PEA)_2_SnI_4_	N/A	3.16	30	NM_T_ 70%	40	N/A	[99]
In_2_O_3_	CNT	2.8	8.6	11.5	NM_H_ 82% NM_L_ 75%	0.8	9700	[109]
IGZO	CNT	12.9	11.7	108.3	N/A	20	N/A	[110]
GZTO	Pentacene	1.2	0.4	52	N/A	8	N/A	[118]
IGZO	F8T2	3.2	1.7 × 10^−3^	67	NM_H_ 10.4 V NM_L_ 18.3 V	30	N/A	[122]

^α^ Total noise margin. ^β^ High noise margin. ^γ^ Low noise margin.

**Table 4:** Materials properties and preparation process of various semiconductor materials.

Materials	Family	Mobility (cm^2^/V·s)	Conduction Type	Preparation Method	Band Gap (eV)	Material Thickness (nm)	Ref.
pentacene	Organic semiconductor	0.718	*p*-type	Organic molecular beam deposition	N/A	50	[46]
MoS_2_	TMD	6	*n*-type	Exfoilation	1.8	2	[69]
MoTe_2_	TMD	22.4	*p*-type	Exfoilation	0.94	4	[70]
IGZO	Metal oxide	4.2	*n*-type	DC magnetron sputtering	2.7	50	[70]
IZO	Metal oxide	3.01	*n*-type	Inkjet printing	>3.0	23	[80]
SWCNT	Carbon nanotube	3–5	*p*-type	Inkjet printing	0.67	1.17	[80]
(PEA)_2_SnI_4_	Perovskite	3.16	*p*-type	Spin coating	N/A	N/A	[99]
F8T2	Organic semiconductor	0.0017	*p*-type	Inkjet printing	N/A	50	[122]

## Data Availability

Not applicable.
